# Root growth characteristics and antioxidant system of *Suaeda salsa* in response to the short-term nitrogen and phosphorus addition in the Yellow River Delta

**DOI:** 10.3389/fpls.2024.1410036

**Published:** 2024-06-06

**Authors:** Jinzhao Ma, Xin Xin, Yu Cao, Liying Zhao, Zehao Zhang, Dongjie Zhang, Zhanyong Fu, Jingkuan Sun

**Affiliations:** Shandong Key Laboratory of Eco-Environmental Science for the Yellow River Delta, Shandong University of Aeronautics, Binzhou, China

**Keywords:** nitrogen and phosphorus addition, *Suaeda salsa*, root morphology, stoichiometry, antioxidant enzyme

## Abstract

Human activities have increased nitrogen (N) and phosphorus (P) inputs to the Yellow River Delta and the supply level of N and P affects plant growth as well as ecosystem structure and function directly. However, the root growth, stoichiometry, and antioxidant system of plants in response to N and P additions, especially for herbaceous halophyte in the Yellow River Delta (YRD), remain unknown. A field experiment with N addition (0, 5, 15, and 45 g N m^-2^ yr^-1^, respectively) as the main plot, and P addition (0 and 1 g N m^-2^ yr^-1^, respectively) as the subplot, was carried out with a split-plot design to investigate the effects on the root morphology, stoichiometry, and antioxidant system of *Suaeda salsa*. The results showed that N addition significantly increased the above-ground and root biomass as well as shoot-root ratio of *S. salsa*, which had a significant interaction with P addition. The highest biomass was found in the treatment with 45 g N m^-2^ yr^-1^ combined with P addition. N addition significantly increased TN content and decreased C:N ratio of root, while P addition significantly increased TP content and decreased C:P ratio. The main root length (MRL), total root length (TRL), specific root length (SRL), and root tissue density (RTD) of *S. salsa* root were significantly affected by N addition and P addition, as well as their interaction. The treatments with or without P addition at the 45 g N m^-2^ yr^-1^ of N addition significantly increased the superoxide dismutase (SOD), peroxidase (POD), catalase (CAT) activities and soluble protein content of roots, decreased malondialdehyde (MDA) content. And there was a significant interaction between the N and P addition on SOD activity. Therefore, N and P additions could improve the growth of *S. salsa* by altering the root morphology, increasing the root nutrient content, and stimulating antioxidant system.

## 1 Introduction

Increased nitrogen (N) deposition caused by human production and activity has been an important factor that can impact the growth and development of plants, thus changing the structure and function of entire ecosystem (Liu et al., 2020). Although there may be positive, negative or neutral effects of such alterations (Xiao et al., 2022), it is widely accepted that N enrichment can increase the aboveground net primary productivity in terrestrial ecosystems, farmland, grasslands, and wetlands (Guan et al., 2019). The improvement of N availability can also increase soil phosphorus (P) availability and plant P concentration, through the stimulation of soil P activity by N. In addition, in some areas, anthropogenic changes in P availability are also increasing, especially in ecosystems receiving sediment input (Lü et al., 2015). The availability of soil N and P directly affects plant N and P concentrations and stoichiometric ratios (Li et al., 2019; Chen et al., 2020). In terrestrial ecosystems, N and P are two major elements that often restrict plant metabolic reaction and growth, and the N:P stoichiometry of plant reflects the biochemical limitations in biological activities such as protein and ribosomal RNA (Lü et al., 2015). Therefore, it is critical to understand how N and P enrichment affect the growth of plants independently and interactively, thus affecting the ecosystem function (Zhan et al., 2017).

The root system is a key organ for land plants to fix in soil and absorb water and mineral nutrients effectively (Sun et al., 2022), and also plays a critical role in the carbon (C) and N cycling (Li et al., 2015). The root structure describes the shape and spatial layout of the root system in the soil. The spatial distribution and morphological characteristics of plant roots have a certain degree of plasticity under the influence of habitat changes and genetic factors (Benlloch-González et al., 2017). Special root structures can be formed to enhance the absorption capacity of nutrients and water, and enhance the survival ability of plants (Li et al., 2020). Atmospheric N deposition can significantly affect the plant root system by changing soil available N and the absorption and transportation of N through leaves (Meng et al., 2021). The chemical, morphological, and physiological characteristics of fine root are sensitive to N application, and the increased soil N availability can also alter the C:N ratio, structure, and activity of root, thus affecting the belowground biomass of plants (Li et al., 2015). In addition, the increase in soil N availability can lead to limitation of other nutrients, such as P (Liu et al., 2020). Increased input of P also could affect the plant root system by influencing root architecture (diameter and length, etc.), biomass, and C cycle functions such as root respiration (Liu et al., 2021; Qian et al., 2023). However, the response of root morphological trait is a complex process. N and P were coupled and influenced by each other, which is important to regulate nutrient limitation and strategies for nutrient uptake in the environment (Zhang et al., 2018). Therefore, exploring the response of plant roots to N and P nutrients is necessary to understand the overall response of underground ecosystem processes to environmental changes.

The Yellow River Delta (YRD), located at the junction of land, river and sea, is a coastal wetland with unique ecosystem and important ecological functions (Zhao et al., 2020). Severe soil salinization ultimately leads to soil ecological degradation and vegetation habitat destruction in the YRD. The typical vegetation in the YRD is dominated by halophytes, which play an important role in soil desalination and restoration (Zhao et al., 2022). *S. salsa* is an annual true halophyte that grows in saline soil and is an ideal species for salt tolerance research (Wang et al., 2021). As a pioneer plant, *S. salsa* shows strong tolerance to environmental stresses such as high salinity and flooding, because of its special structure and ion transport system (Wu et al., 2012; Guo et al., 2019). *S. salsa* also plays an important role in improving soil quality, reducing coastal erosion, regulating climate and improving habitat quality in the YRD (Chen & Sun, 2020; Wang et al., 2021). It has been found that the N deposition has been as high as 22.64 kg hm^-2^ in the growing season in the YRD, which is almost one of the highest N deposition areas in China (Guan et al., 2019). In recent years, the effects of N deposition on coastal wetland ecosystems has received increasing attention, mainly involving studies on the growth and ecological characteristics of plants, soil physicochemical properties, soil microbial community diversity, etc (Guan et al., 2019; Lu et al., 2021). However, the effects of exogenous N enrichment on the root growth of regional halophyte remain unknown. It is necessary to study of the effects of N and P addition on the growth and antioxidant system of halophyte root in coastal wetland of the YRD in order to the comprehend the response of coastal wetland ecosystem functions to human activities.

In this paper, the field *in situ* N and P addition experiment of the YRD was conducted and the effects of exogenous nutrient enrichment on variation of *S. salsa* root were studied. The objectives of the study were: 1) dose N and P addition have an impact on the root growth characteristics of *S. salsa*? 2) how do nutrient contents of root respond to different levels of N and P addition? 3) how do N and P affect antioxidant enzyme activities of the *S. salsa* root system in a saline wetland ecosystem? The results could provide insight into the response of wetland plants to environmental stresses and give valuable implications for wetland vegetation restoration in the Yellow River Delta.

## 2 Materials and methods

### 2.1 Study site

**Figure 1:**
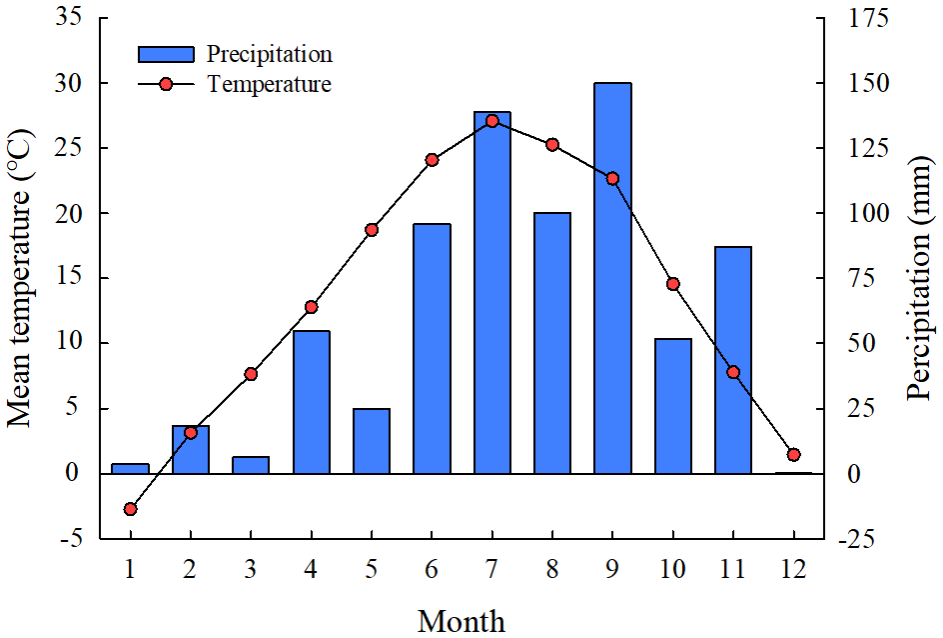
Monthly mean temperature and precipitation of the experimental site in 2021.

The experimental site was located in the Yellow River Delta of Dongying City, Shandong province, China (N 37°55’26’’, E 118°34’37’’), which belongs to the warm temperate monsoon continental semi-humid climate zone. The mean annual temperature is 12.1°C, the mean annual precipitation is 550 mm, which 70% of rainfall concentrated between July and September (**Figure 1**), and the annual evaporation capacity is about 1900–2400 mm in the experimental area (Lu et al., 2021). The typical vegetation is mainly *Phragmites australis*, *S. salsa*, *Tamarix chinensis*, and *Aeluropus sinensis*, which mostly growing in sandy and argillaceous soils with poor nutrient conditions. The soil characteristics of the top 20 cm soil in the study sites include a pH of 7.83, electrical conductance (EC) of 5.38 ms·cm^-1^, organic matter (OM) 16.2 g kg^-1^, total N (TN) 0.32 g kg^-1^, total P (TP) 0.64 g kg^-1^, total potassium (TK) 0.64 g kg^-1^, and soil salt content 16.2 g kg^-1^.

### 2.2 Experimental design

The experiment was conducted in 2021 with a randomized block design (2 m × 2 m plots which were separated by 2 m buffers to prevent the interference caused by fertilizer movement among the experimental plots). The study was a multifactorial experiment considering N and P addition levels as two nutrient factors, including four levels of N fertilization (N0, N5, N15, and N45 was 0, 5, 15, and 45 g N m^-2^ yr^-1^, respectively) and two levels of P fertilization (P0 and P1 was 0 and 1 g P m^-2^ yr^-1^, respectively). The experiment consisted of eight treatments: N0P0, N5P0, N15P0, N45P0, N0P1, N5P1, N15P1, and N45P1, with three replicate plots. A total of 24 plots were conducted and the determination of the N and P application rates was based on previous study in the YRD (Guan et al., 2019; Li et al., 2019).

N fertilizer in the form of urea (N 46%) and P fertilizer in the form of KH_2_PO_4_ (Li et al., 2019), were dissolved in 4 L deionized water and sprayed uniformly to each plot using a sprayer (Wang et al., 2019). The whole treatments were applied once a month from May 2021 to August 2021 and the control treatment (N0P0) was sprayed with equal amounts of deionized water. The cumulative value of 4 months reached the processing value.

### 2.3 Sample preparation and measurements

We randomly sampled 10 live complete plants at an area of 0.5 m × 0.5 m within each plot in late August. The whole plants were collected by using the full excavation method to maintain the integrity of root system during the process (Sun et al., 2022). All the samples were placed in a cold storage box (4°C) and taken to the laboratory immediately for the further analysis.

The roots were washed with tap water carefully to remove the attached soil and rinsed thoroughly with distilled water again. Then the aboveground and belowground (root) parts were separated and one part of them were oven-dried at 105°C for 2 hour and then 75°C for 48 h to a constant weight (Li et al., 2021), and the other part were stored in a refrigerator at 4°C for the detection of antioxidant system. The aboveground biomass and root biomass were measured, and the root-shoot ratio = root biomass/aboveground biomass × 100%. Furthermore, the root samples were scanned with an Epson digital scanner and analyzed with a WinRHIZO professional root analysis system. The main root length (MRL), total root length (TRL), surface area, volume, and average diameter (AD) were measured. And the measurements were used to calculated: Specific root length (SRL, cm g^-1^) = root length/dry weight, specific root surface area (SRA, cm^2^ g^-1^) = surface area/dry weight, and root tissue density (RTD, g cm^-3^) = dry weight/volume were calculated (Zhu et al., 2021).

The oven-dried root samples were grounded with a ball mill and passed through a 0.1 mm sieve for the element analysis (Zhu et al., 2021; Xiao et al., 2022). The total carbon (TC) and TN concentrations of root were analyzed using an elemental analyzer (vario MACRO CUBE, Elementar, German). TP concentration of root samples was determined with the molybdate/ascorbic acid method after the wet digestion with H_2_SO_4_-H_2_O_2_ (Zhan et al., 2017; Zhang et al., 2018). The ratios of C:N, C:P, N:P were calculated with previous studies (Li et al., 2017b).

Three soil samples were randomly collected using a 5 cm-diameter soil drill at 0–20 cm depths from each plot, and combined as a single composite sample on the same day. All the fresh soil samples were taken back to the laboratory and extracted with 2 mol L^-1^ KCl, then the solution was determined using a follow injection autoanalyzer (AA3, SEAL, USA) for measuring ammonium (NH_4_^+^-N) and nitrate (NO_3_^−^-N) contents (Zhan et al., 2017). Then the samples were air-dried, ground, and sieved through a 2 mm mesh and a 0.25 mm mesh, respectively, for the determination of the following indicators. Soil pH was determined with a 1:5 (soil: water, w/v) solution by a digital pH meter (FE22-Standard, Mettler Toledo, USA). Soil salt content was measured by the weight loss of 1:5 (soil: water, w/v) extract solution after oven-drying to a constant weight at 105°C (Guan et al., 2019). Soil organic matter (OM) was measured by the potassium dichromate-sulfuric acid oxidation method (Liu et al., 2020). Soil available P (AP) was extracted with the 0.5 mol L^-1^ NaHCO_3_ and the leaching solution was measured ammonium molybdate method (Li et al., 2017b).

For antioxidant system, 0.5 g frozen root samples per replication were crushed and homogenized using a 50 mM potassium phosphate buffer (pH 7.0), containing 1% polyvinyl pyrrolidone and 1 mmol L^-1^ EDTA-Na_2_. The homogenate was centrifuged at 12,000×g for 20 min at 4°C, and then the supernatant solution was used in following assays (Aghaleh et al., 2011). Superoxide dismutase (SOD) activity was determined by the reaction of nitroblue tetrazolium using the riboflavin system under the illumination as described by Guo JM (Guo et al., 2021). Peroxidase (POD) activity was measured in the reaction mixture containing 50 mmol phosphate buffer (pH 7.0), 19 μl H_2_O_2_, 28 μl guaiacol, and 100 μl of enzyme extract at 430 nm (Zhu et al., 2020). Catalase (CAT) activity was determined by monitoring the consumption of H_2_O_2_ from the reaction mixture at 240 nm for 3 min (Ibrahim et al., 2020). Malondialdehyde (MDA) content was determined by the thiobarbituric acid reaction as described by (Azizi et al., 2022). The mixture was heated in a water bath at 98°C for 20 min, then cooled and centrifuged at 4000 × g for 20 min. The supernatant was measured at 600, 532, and 450 nm. Soluble protein (SP) concentration was determined by the Coomassie Brilliant Blue G-250 dye-binding method (Ma et al., 2019).

### 2.4 Data analysis

One-way ANOVA with the LSD test was used to analyze the differences in biomass, root-shoot ratios, root growth indicators (MRL, TRL, AD, SRL, SRA, and TD), nutrient concentrations (TC, TN, TP, and C:N:P ratios), antioxidant enzyme (SOD, POD, CAT) activities, MDA and SP content of *S. salsa* root, and soil properties and fertility (pH, salt content, TC, TN, TP). Two-way ANOVA with Duncan tests was used to examine the effects of N, P and their interactions on the indicators. The data analyses were conducted with SAS 9.2 (2010, SAS Institute Cary, NC) and were expressed as the mean ± standard error (n=3). Difference was scores as significant at the *P*<0.05 levels. The figures were generated using SigmaPlot 12.0, and the Pearson correlation and principal component analysis (PCA) were performed using R software (4.1.3) by Corr plot (0.92) package and prcomp function.

## 3 Results

### 3.1 Effects of different treatments on the biomass of *S. salsa*

**Figure 2:**
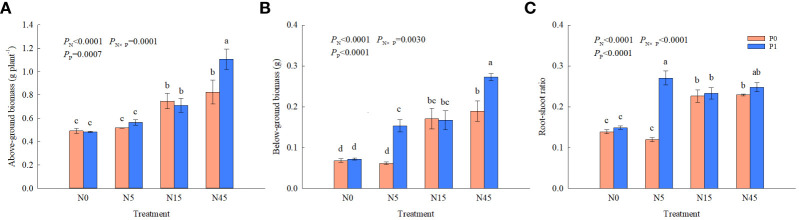
Biomass **(A, B)** and root-shoot ratios **(C)** of *S. salsa* under different treatments. Different lowercase letters show significant differences (*P*<0.05). The same as below.

There were significant effects of N, P and their interaction on above-ground biomass, below-ground biomass and root-shoot rations (**Figure 2**). N15 and N45 treatments significantly increased above-ground biomass and root biomass than N0 treatment by 52.0~67.7% and 151.5~177.9%, respectively, in the absences of P. In comparison to the other treatments, N45 treatment resulted in 55.4~128.7% and 63.5~279.2% higher above-ground biomass and below-ground biomass, respectively, when P was added. When the N addition was 0~15 g N m^-2^ yr^-1^, there was no effect on above-ground biomass with or without P addition. However, above-ground biomass and root biomass in N45P1 treatment were increased significantly than that in N45P0 treatment. The N1P1 treatment resulted in 94.2% and 125.0% higher root-shoot ratio than N0P0 and N0P1 treatments, respectively. When N addition was 0, 15, and 45 g N m^-2^ yr^-1^, there was no significant difference in root-shoot ratio between P0 and P1 treatments.

### 3.2 Effects of different treatments on the root morphology of *S. salsa*

The MRL, TRL, AD, SRL, and RTD of *S. salsa* root were significantly affected by N addition and P addition, as well as their interaction except AD of root (**Table 1**). MRL was increased significantly with the increasing N addition whether P was added or not. N45 treatment resulted in the highest TRL with or without P addition. When the N addition was 5 and 45 g N m^-2^ yr^-1^, the P addition significantly increased MRL, TRL, AD than the treatment without P addition.

**Table 1:** Root morphology of *S. salsa* under different treatments.

Treatment	MRL (cm)	TRL (cm)	AD (mm)	SRL (cm g^-1^)	SRA (cm^2^ g^-1^)	RTD (g cm^-3^)
N0P0	4.43 ± 0.12e	23.17 ± 2.60e	0.13 ± 0.01d	304.48 ± 12.17c	50.7 ± 7.21b	2.33 ± 0.39bc
N5P0	6.07 ± 0.23d	25.91 ± 1.23e	0.22 ± 0.01d	418.58 ± 19.87a	71.05 ± 11.53a	0.85 ± 0.10de
N15P0	7.15 ± 0.26c	42.11 ± 3.34c	0.45 ± 0.07bc	263.99 ± 9.23d	38.07 ± 9.79c	2.27 ± 0.22bc
N45P0	8.27 ± 0.42b	58.79 ± 4.85b	0.54 ± 0.02b	310.49 ± 25.62c	52.68 ± 4.55b	1.44 ± 0.15cd
N0P1	4.47 ± 0.26e	29.90 ± 1.91d	0.26 ± 0.02d	389.08 ± 3.70b	33.19 ± 2.6c	2.66 ± 0.48b
N5P1	7.35 ± 0.26c	41.41 ± 2.20c	0.39 ± 0.04c	270.08 ± 14.33d	30.22 ± 6.76c	4.54 ± 0.27a
N15P1	8.20 ± 0.42b	40.72 ± 3.59c	0.51 ± 0.01bc	243.34 ± 21.46d	51.30 ± 7.02b	1.87 ± 0.30bc
N45P1	9.70 ± 0.23a	71.29 ± 1.84a	0.83 ± 0.08a	261.62 ± 6.75d	78.67 ± 13.25a	0.28 ± 0.01e
N	**	**	**	**	**	**
P	**	**	**	**	NS	**
N×P	*	**	NS	**	**	**

MRL, main root length; TRL, total root length; AD, average diameter; SRL, specific root length; SRA, specific root surface area; RTD, root tissue density. Different lowercase letters in the same column show significant differences (*P*<0.05). *, *P* ≤ 0.05; **, *P* ≤ 0.01; NS, not significantly. The same as below.

The SRL and SRA in N5 treatment were increased significantly than other treatments in the absence of P. There was no significant difference of SRL among the N5P1, N15P1 and N45P1 treatments. SRA with P addition was increased significantly than that without P addition when the N addition was 15 and 45 g N m^-2^ yr^-1^. N5P1 significantly increased RTD by 434.1%, 70.7%, and 142.8%, respectively, compared with N5P0, N0P1, and N15P1 treatment.

### 3.3 Effects of different treatments on the ecostoichiometry of *S. salsa* root

**Figure 3:**
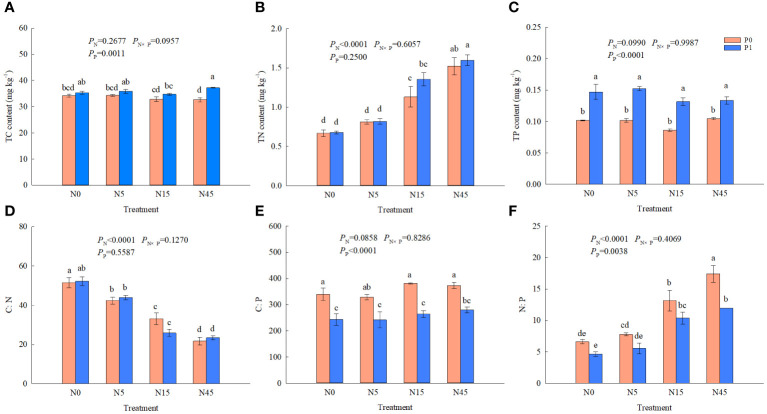
TC **(A)**, TN **(B)**, TP **(C)** concentrations and C:N **(D)**, C:P **(E)**, N:P **(F)** ratios of *S. salsa* root under different treatments. (*P*<0.05). Different lowercase letters in the same column show significant differences (P<0.05).

N addition significantly affected TN, C:N and N:P ratio of *S. salsa* root and P addition significantly affected TC, TP, C:P, and N:P ratio. While it was not significantly affected by N and P interaction on the nutrient contents and C:N:P ratios (**Figure 3**). N45P1 treatment increased TC content by 45.5% and 14.1% compared with N15P1 and N45P0 treatments, respectively. No significant difference was found in TC content among the N treatments without the P addition. The TN content was increased with increasing N addition rate with or without P addition. Under the same N addition, there was no significant effect on TN content and C:N ratio between P1 and P0 treatment. The treatment with P addition increased significantly TP content of root compared with no P addition. C:N ratio was decreased significantly with increasing N addition rate and P addition treatment significantly reduced C:P ratio under the same N addition. However, no significant differences were found in TP content and C:P ratio among the different N treatments. The highest N:P ratio was obtained from the N3P1 treatment, which was higher 32.2~274.0% than all the other treatments.

### 3.4 Effects of different treatments on the root antioxidant system of *S. salsa*

**Figure 4:**
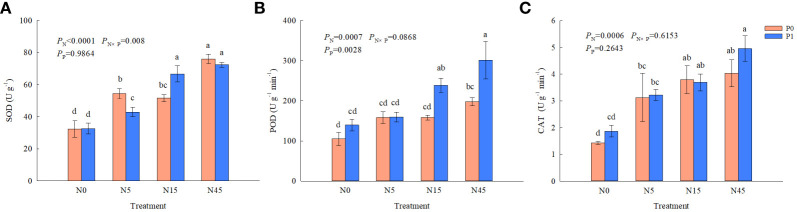
Antioxidant enzyme **(A–C)** activities of *S. salsa* root under different treatments. (*P*<0.05). Different lowercase letters in the same column show significant differences (P<0.05).

There were significant effects of N addition on SOD, POD, CAT activities and P addition on POD activities, whereas the interaction of N and P addition only significantly affected SOD activities (**Figure 4**). N addition significantly improved SOD activities compared with N0 treatment with or without P addition. The highest POD activity was obtained from the N45P1 treatment, which was higher 52.5% and 115.7% than N45P0 and N0P1, respectively. There was no significant difference between P1 and P0 treatment when the N addition was 0~5 g N m^-2^ yr^-1^. The N addition treatments improved CAT activities significantly compared with N0 treatment when no P addition, whereas no significant difference was found between P0 and P1 treatments with the same N addition.

**Figure 5:**
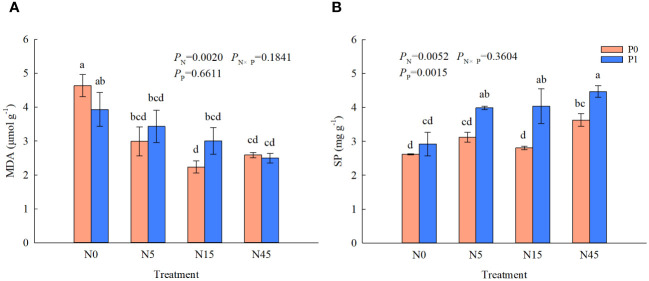
MDA **(A)** and SP **(B)** content of *S. salsa* root under different treatments. (*P*<0.05). Different lowercase letters in the same column show significant differences (P<0.05).

The MDA contents of *S. salsa* root were significantly affected by N addition (**Figure 5**). Whether P is added or not, N5, N15 and N45 treatments decreased the MDA content significantly compared with N0 treatment. However, the MDA content was not significantly affected by P addition. There were significant effects of N addition, and the interaction of N and P on the soluble protein of *S. salsa* root. N addition (N5, N15, and N45 treatments) significantly increased the soluble protein by 52.3~70.6% than N0 treatment with P addition. The SP contents in the treatments with P addition were increased significantly than that without P addition when the addition was 5~45 g N m^-2^ yr^-1^.

### 3.5 Effects of different treatments on soil properties and fertility

There were significant effects of N addition on soil salt, NH_4_^+^-N, and NO_3_^–^N content, whereas the interaction of N and P addition only significantly affected soil organic carbon (**Table 2**). The soil salt contents in N45P0 and N45P1 treatments were decreased by 29.6% and 26.1% than that in N0P0 treatment, respectively. Compared with N0P0 treatment, N5P0, N15P0 and N45P0 treatments significantly increased NH_4_^+^-N content by 46.9~94.4% and NO_3_^–^N content by 59.1~169.36%, respectively. N15P1 and N45P1 treatments significantly increased NH_4_^+^-N content than N0P1 treatment by 49.4~70.2%, NO_3_^–^N content by 70.1~203.8%. There was no significant effect on soil NH_4_^+^-N and NO_3_^–^N content with or without phosphorus addition at the same N addition level. Soil AP content was significantly affected by P addition. The N0P1, N15P1, and N45P1 treatments significantly increased AP content by 55.8~72.2% compared with N0P0 treatment. All the treatments showed no significant difference in soil pH and OM content.

**Table 2:** Soil properties and fertilizer under different treatments.

Treatment	pH	Salt content (‰)	OM (g kg^-1^)	NH_4_^+^-N (mg kg^-1^)	NO_3_^–^N (mg kg^-1^)	AP (mg kg^-1^)
N0P0	7.41 ± 0.14a	11.30 ± 0.30a	12.73 ± 1.74a	4.14 ± 0.35d	11.88 ± 0.84e	16.52 ± 3.56c
N5P0	7.34 ± 0.07a	10.50 ± 0.48ab	12.02 ± 1.79a	6.08 ± 0.47bc	18.90 ± 0.73bc	21.31 ± 0.32abc
N15P0	7.37 ± 0.09a	9.13 ± 0.50bc	21.20 ± 1.97a	7.41 ± 0.40ab	22.63 ± 0.22b	18.56 ± 2.98bc
N45P0	7.32 ± 0.03a	7.95 ± 0.78c	16.28 ± 1.19a	8.05 ± 0.60a	32.00 ± 2.08a	19.80 ± 2.02bc
N0P1	7.42 ± 0.06a	11.47 ± 0.23a	19.71 ± 1.35a	4.29 ± 0.16d	10.89 ± 0.80e	26.69 ± 4.19ab
N5P1	7.44 ± 0.04a	11.83 ± 0.67a	18.69 ± 1.50a	5.43 ± 0.25cd	15.59 ± 1.93cd	23.65 ± 0.29abc
N15P1	7.40 ± 0.04a	10.58 ± 0.96ab	17.03 ± 1.89a	6.41 ± 0.51bc	18.62 ± 1.28c	28.45 ± 1.27a
N45P1	7.31 ± 0.03a	8.35 ± 0.78c	15.08 ± 1.62a	7.30 ± 0.60ab	33.08 ± 1.11a	25.73 ± 2.02ab
N	NS	**	NS	**	**	NS
P	NS	NS	NS	NS	NS	*
N×P	NS	NS	NS	NS	NS	NS

OM, organic matter; AP, available phosphorus. Different lowercase letters in the same column show significant differences (*P*<0.05). *P* ≤0.05; **, *P* ≤ 0.01; *, *P* ≤ 0.05; NS, not significantly.

### 3.6 Relationship among soil property, root morphology, nutrient content, and antioxidant system of *S. salsa* root

**Figure 6:**
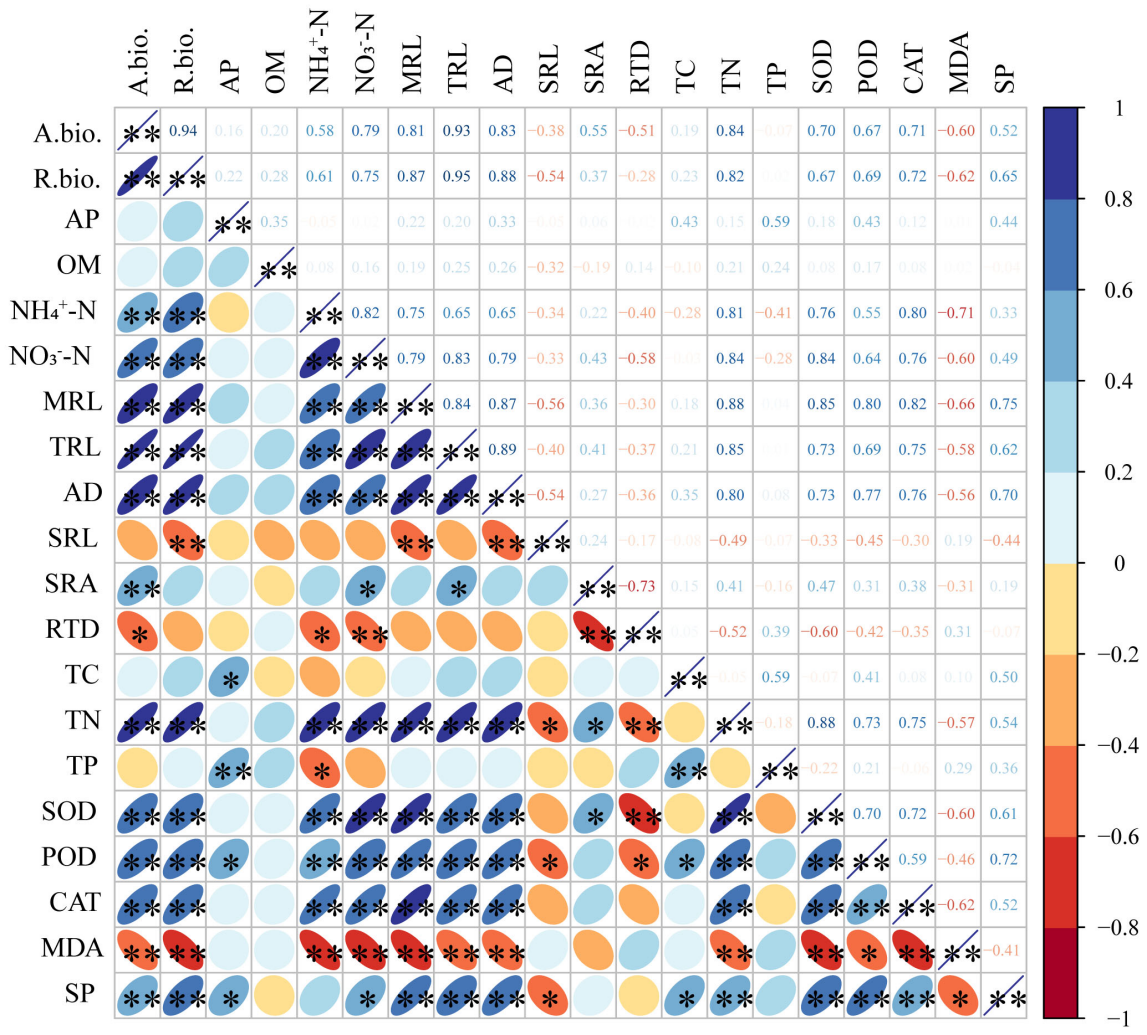
Correlation analysis of the *S. salsa* root system indexes and soil property. *, *P ≤* 0.05; **, *P ≤* 0.01; A.bio., aboveground biomass; R.bio., root biomass; OM, organic matter; AP, available phosphorus; NH_4_^+^-N, ammonium; NO_3_^−^-N, nitrate; MRL, main root length; TRL, total root length; AD, average diameter; SRL, specific root length; SRA, specific root surface area; RTD, root tissue density; TC, total carbon; TN, total nitrogen; TP, total phosphorus; SOD, superoxide dismutase; POD, peroxidase; CAT, catalase; MDA, malondialdehyde; SP, soluble protein.

Significant correlations were found among growth characteristics, nutrient concentration, antioxidant system of *S. salsa* root and soil property and fertility (**Figure 6**). The TN of root was positively correlated with soil NH_4_-N and NO_3_-N content, MRL, TRL and AD of root (*P*<0.01), SRA (*P*<0.05), and negatively correlated with soil salt content, RTD (*P*<0.01), SRL (*P*<0.05). The TP content of root was positively correlated with soil AP content, root TC content (*P*<0.01), and negatively correlated with soil NH_4_-N content (*P*<0.05). SOD, POD, and CAT activities were positively correlated with soil NH_4_-N, NO_3_-N content, MRL, TRL, AD and TN content of root (*P*<0.01). However, the correlation of MDA content was opposite to that of SOD, POD, and CAT activity. SP content was positively correlated with soil NO_3_-N and AP content, root TC content (*P*<0.05), MRL, TRL, AD, root TN content (*P*<0.01), and negatively correlated with SRL and MDA content (*P*<0.05).

**Figure 7:**
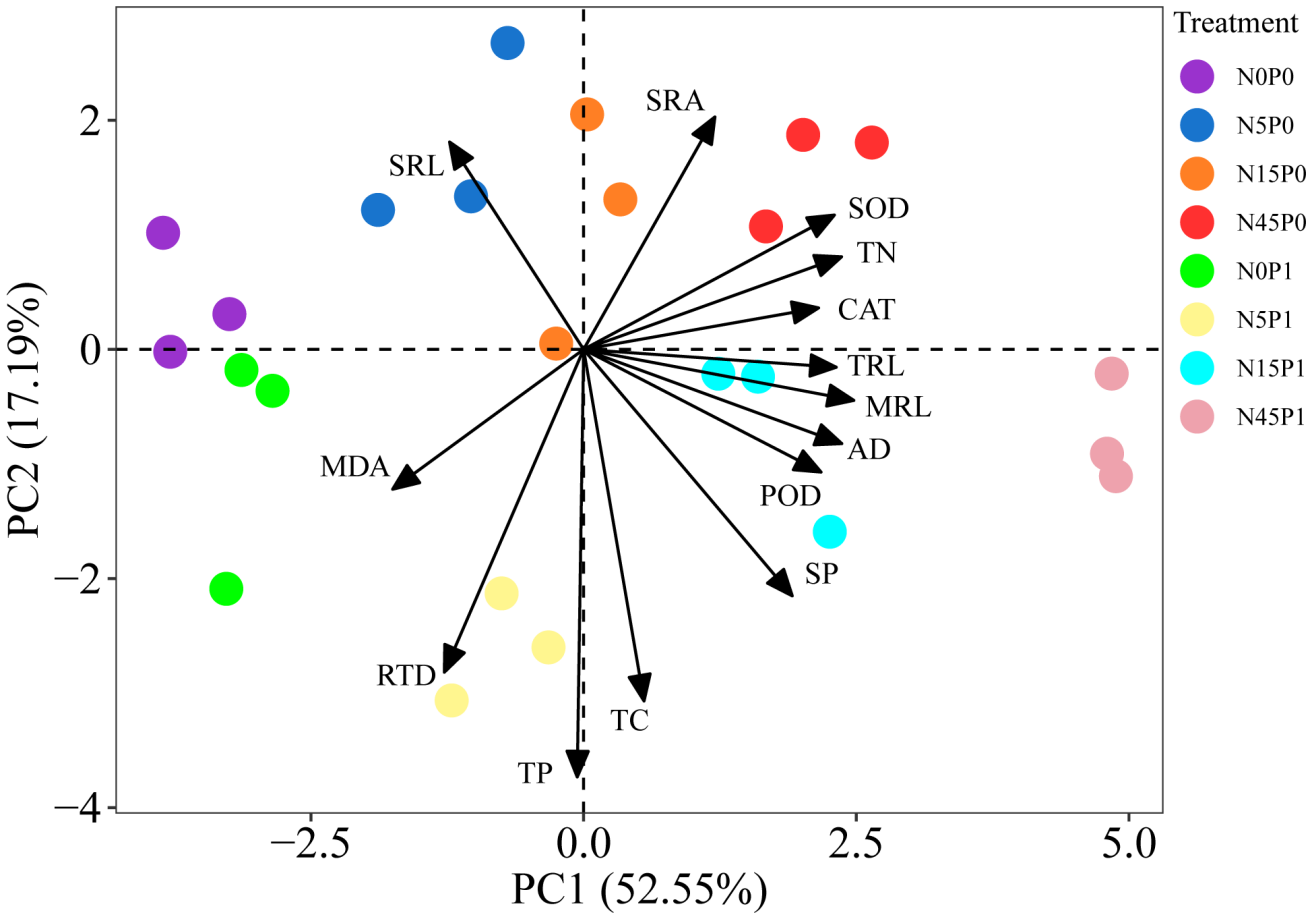
PCA of nutrient content, root morphology, and antioxidant system of *S. salsa* root. MRL, main root length; TRL, total root length; AD, average diameter; SRL, specific root length; SRA, specific root surface area; RTD, root tissue density; TC, total carbon; TN, total nitrogen; TP, total phosphorus; SOD, superoxide dismutase; POD, peroxidase; CAT, catalase; MDA, malondialdehyde; SP, soluble protein.

The PCA revealed that the two major PC components together explained 69.74% of the data variability, which could reflect the expression information of the 14 indicators well (**Figure 7**). TRL, MRL, AD, TN and SP content, and antioxidant enzyme activity of *S. salsa* root had positive loads in the PC1, which explain 52.55% of the data variability. The negative load of TP, TC content and RTD and the positive load of SRA and SRL were all found in the PC2, which explained 17.19% of the observed variability. The N45P1, N45P0, and N15P1 treated groups were clearly separated from N0P0, N0P1 treated groups along PC1. N15P0 and N5P0 treated groups were separated from N5P1 treated group along PC2.

## 4 Discussion

### 4.1 Effects of N and P addition on the biomass and root characteristics of *S. salsa*

The allocation of biomass is the result of long-term adaptation of plants to the natural environment, which could maximize the use of environmental conditions (Wang et al., 2023). Plant biomass allocation strategies will change in response to the environmental conditions (Sugiura et al., 2016). The meta-analysis of Li et al. (2015) showed that simulated N deposition significantly increased total root biomass by 20.2%. In this experiment, N addition of 15–45 g N m^-2^ yr^-1^ significantly increased aboveground and root biomass of *S. salsa*. There was no significant difference in aboveground biomass with phosphorus application at the N addition of 0–15 g N m^-2^ yr^-1^, while P application significantly increased aboveground and belowground biomass of the plant at 45 g N m^-2^ yr^-1^ addition. This suggested that N application stimulates plant growth and increases its demand for phosphorus (Li et al., 2016). Root-shoot ratio is an important parameter in the study of the spatial distribution of plant biomass and carbon stocks, which exhibits a high degree of plasticity to adapt to the external environment in order to optimize the allocation of resources (Sun et al., 2022). N addition (15–45 g N m^-2^ yr^-1^) was found to significantly increase the root-shoot ratio of *S. salsa* in our study. Allocating more biomass to underground part could maintain a higher root-shoot ratio, increasing more nutrients and water to the plant and reducing the transpiration surface area (Sugiura et al., 2016). And the high biomass allocation of below-ground organs enables greater functioning of the below-ground root system (Sainju et al., 2017). We also observed that the interaction of N and P significantly increased biomass and root-shoot ratio. The findings suggested that continued changes in human-caused inputs of N and P will significantly alter the dynamics of terrestrial biomass.

Root morphological traits are highly plastic in response to environmental changes, and the root changes are related to individual nutrient requirements and nutrient stress (Qian et al., 2023). The results of ANOVA showed that the root morphology of *S. salsa* was not only affected by single N or P addition, but also by the interaction of N and P addition. MRL and TRL of *S. salsa* were significantly increased by nitrogen addition, which suggested that increasing the root length of herbaceous plants can improve the efficiency of N uptake in soils under N application conditions (Wang et al., 2023). P application was able to significantly promote its growth with N addition of 5–45 g N m^-2^ yr^-1^, while the effect of P application without N addition was not significant. This showed that the addition of N leads to extensive P restriction (Gonzales & Yanai, 2019). SRL, SRA and RTD are relevant physiological properties of the root system, reflecting nutrient acquisition by the root system and plant growth strategies (Ahmadi et al., 2018). N0P1 treatment had higher SRL and RTD than N45P1 treatment, suggesting that plants could acquire more nutrients by altering root morphology under nitrogen deficiency. In addition, plants can optimize C inputs by controlling the AD of roots, thereby increasing SRL, which is consistent with our findings. Variation in the root morphology between the increasing N input effects on *S. salsa* perhaps because that root-surface phosphomonoesterase activities was stimulated by N addition, which enhances P conservation and accelerates P cycling rates (Li et al., 2019). Secondly, the input of N and P may alter the root traits by increasing plant nutrient absorption and nitrates and phosphates are both signaling molecules that stimulate root branching (Schleuss et al., 2020). Root morphology not only determines the spatial distribution characteristics of the root system, but also has an important impact on the nutrient uptake capacity of the root system as well as its immobilization, which can reflect the feeding strategy of the root system under different habitat conditions (Li et al., 2017a).

### 4.2 Effects of N and P addition on the root ecostoichiometry of *S. salsa*

C, N, and P contents of plant organs are important indicators of the plant nutritional status, which could reflect the adaptation of plants to environmental condition (Zhao et al., 2022). In our study, short-term N addition (15–45 g N m^-2^ yr^-1^) increased the TN content of *S. salsa* roots, which was positively correlated with above-ground and root biomass of the plant (*P*<0.01). However, P additions had no significant effect on TN of the roots at the same nitrogen levels. Previous studies have shown that the appropriate N addition promotes N accumulation and C fixation and then increase the plant life activities, resulting in the accumulation of more nutrients (Liu et al., 2020). We found that P addition significantly increased the TP content of the *S. salsa* root, while N addition had no significant effect on TC and TP of the root. This may be due to the short duration of N and P additions and the fact that roots are more insensitive to changes in NP content compared to leaves of plant (Zhan et al., 2017). Combined addition of N and P, the organic P storage, P recycling, and plant P absorption increased with the level of N addition. The addition of N stimulates the absorption of P by plants when the inorganic phosphorus is sufficient, and leads to the depletion of soil dissolved inorganic P (Schleuss et al., 2020). Plants can adjust their nutrient uptake strategies according to the amount of N and P of the environment, which affects their photosynthetic and carbon partitioning capacity, leading to changes in nutrient content and ratios among their root systems (Li et al., 2021).

The stoichiometric ratios of plant tissues can reflect the uptake and utilization of elements by plants to some extent, varying with their growth status and environmental conditions (Liu et al., 2020). The study showed that short-term N and P additions had significant effects on the C:N:P stoichiometric characteristics of *S. salsa* root. N addition significantly reduced the C:N ratio and increased N:P in the root system, which is consistent with the study by Lei Li (Li et al., 2019). Without P addition, N:P ratio of root ranged from 6.6 to 17.4 with increasing N level, suggesting that root growth was significantly N-limited without N addition, but gradually shifted to P-limited with the N addition. Plants can utilize N to up-regulate P uptake and transport systems. Because the dissolved inorganic P content in plant cells is higher than that in soil and plants use proton-ATPase to transport phosphate across the plasma membrane. The addition of N can increase the activity of anion/H+ co-transport pumps, as NH_4_^+^ requires the release of H^+^ (Schleuss et al., 2020). P is a limiting nutrient element for plant growth in the YRD, and the reason for P limitation in halophytes is mainly related to the effectiveness of soil P. The soil in the region, subjected to seawater erosion, is mainly by the calcium adsorbed form of P, which is more stable and hard to be absorbed by plants (Zhao et al., 2022). P addition could be effective in alleviating the P limitation caused by the increased N.

### 4.3 Effects of N and P addition on the root antioxidant system of *S. salsa*

Soil salinization in the YRD has increased due to insufficient groundwater replenishment, continuous seawater intrusion and high evapotranspiration (Guan et al., 2019; Zhao et al., 2020). Massive increase of reactive oxygen species (ROS) in plants causes severe damage to the organism under salt stress (Azizi et al., 2022). The plant reduces the damage produced by ROS through increasing the activity of antioxidant enzymes, such as SOD, POD, and CAT et al (Zhu et al., 2020). The results of our study showed that N addition significantly increased SOD, POD and CAT enzyme activities of *S. salsa* root compared to no N addition and there was a significant positive interaction on SOD activity between N and P addition. POD and CAT can effectively reduce H_2_O_2_ decomposed from O_2_^-^ by SOD with increasing salinity (Aghaleh et al., 2011). P addition significantly increased POD activity, while had no significant impact on CAT activity, at the N addition of 15–45 g N m^-2^ yr^-1^. The enzymes act synergistically to defend against cell membrane damage caused by ROS, inhibit membrane lipid peroxidation, and mitigate the damage caused by salt stress, thus keeping the production and removal of reactive oxygen species in a balanced state (Li et al., 2020; Qian et al., 2023). MDA, as a product of cellular membrane lipid peroxidation, reflects the strength of membrane lipid peroxidation and the degree of membrane lipid damage (Qian et al., 2023). MDA content was significantly reduced with N addition, which could be related to the increase of antioxidant enzyme activity. N addition significantly increased root SP content, and P addition significantly increased SP content under the same N addition level. Correlation analysis revealed that root TN content was positive correlated with root antioxidant enzymes and SP content (*P*<0.01), and negative correlated with MDA content (*P*<0.01). N and P both are the main elements involved in protein synthesis in the process of plant growth, so N and P addition could effectively improve the activity of antioxidant enzymes of *S. salsa*, enhancing the survival ability.

## 5 Conclusion

N and P addition significantly increased aboveground, belowground biomass, and root-shoot ratio of *S. salsa* and there was a significant positive interaction between N and P among them. The root morphology (MRL, TRL, AD, SRL, and RTD) of *S. salsa* varied with the level of N and P addition which promoted the nutrient absorption. N addition significantly affected TN, C:N and N:P ratio and P addition significantly affected TC, TP, C:P, and N:P ratio of *S. salsa* root. N treatment effectively increased antioxidant enzyme activities and SP content of root, while decreased MDA content. When the N addition was 15–45 g N m^-2^ yr^-1^, P addition significantly improved POD activity and SP content. This study suggests that moderate amount of N and P addition could effectively increase the nutrient content and antioxidant enzyme activities of the root system, promote its root development, and then increase its biomass. The results could provide a theoretical support for the response of wetland plants to the environment and vegetation restoration.

## Data availability statement

The original contributions presented in the study are included in the article/supplementary material. Further inquiries can be directed to the corresponding author.

## Author contributions

JM: Writing – original draft. XX: Writing – original draft, Resources, Methodology, Investigation. YC: Writing – original draft, Resources, Investigation. LZ: Writing – original draft, Resources, Investigation. ZZ: Writing – original draft, Resources, Investigation. DZ: Writing – review & editing. ZF: Writing – review & editing. JS: Writing – review & editing.
